# Rapid identification of antigen-specific TCRs for cancer immunotherapy

**DOI:** 10.52601/bpr.2025.250003

**Published:** 2026-04-30

**Authors:** Min Jiang, Wenling Wang, Shuguang Tan

**Affiliations:** 1 Medical School, University of Chinese Academy of Sciences, Beijing 101408, China; 2 CAS Key Laboratory of Pathogen Microbiology and Immunology, Institute of Microbiology, Chinese Academy of Sciences, Beijing 100101, China; 3 Institutes of Physical Science and Information Technology, Anhui University, Hefei 230039, China; 4 The Second Affiliated Hospital, Zhejiang University School of Medicine, Hangzhou 310058, China

**Keywords:** Immunopeptide, Single-cell sequencing, T cell receptor, T cell receptor-engineered-T cell, Immunotherapy

## Abstract

T cell receptors (TCRs) can recognize peptides presented by major histocompatibility complex (MHC) molecules, referred to as HLA in humans, which enables the targeted eradication of tumor cells expressing specific antigens. In recent years, TCR-engineered T cell (TCR-T) cell therapy has demonstrated substantial advancements in clinical trials targeting solid tumors. Notably, in August 2024, the U.S. FDA approved the first TCR-T drug for the treatment of advanced synovial sarcoma, representing a pivotal milestone in the field. For the development of TCR-T therapy, identifying tumor-associated antigen epitopes and high-functional TCRs are critical. Here, we present a comprehensive protocol outlining the process of identification of immunogenic epitopes and the efficient screening of antigen-specific TCRs from HLA transgenic mice. Additionally, the protocol encompasses methodologies for TCR-T cell preparation and their functional evaluation *in vitro*. These approaches provide a robust framework for advancing the development of tumor-specific TCRs and fostering the clinical translation of TCR-T therapies.

## INTRODUCTION

Cancer immunotherapies based on T cells include tumor-infiltrating lymphocyte (TIL) therapy, chimeric antigen receptor T cell (CAR-T) therapy, and T cell receptor-engineered T cell (TCR-T) therapy (Waldman *et al.* 2020), with the latter two involving genetic modification of T cells. CAR-T therapy predominantly targets cell surface antigens, whereas TCR-T therapy exploits the specificity of T cell receptors (TCRs) to recognize tumor-associated antigenic peptides presented by MHC molecules (HLA in humans) (Baulu *et al.* 2023). This distinct mechanism of TCR-T cells broadens the range of targetable antigens, including intracellular tumor-derived proteins (Garber 2018). Such capacity is particularly advantageous for addressing solid tumors, which often lack highly expressed surface antigens. The TCR plays a critical role in initiating immune responses by precisely recognizing antigen peptides and activating T-cell signaling pathways. Upon recognizing tumor-specific antigens, TCR-T cells are rapidly activated, deploying perforin and granzyme to directly induce apoptosis, and secreting cytokines such as IFN-γ and TNF-α to enhance antitumor immunity and modulate the tumor microenvironment (Shah *et al.* 2021).

The clinical application of TCR-T therapy has advanced significantly in recent years. Notably, the FDA has approved afamitresgene autoleucel (Afami-cel; TECELRA®), the first TCR-T therapy for patients with HLA-A2-restricted synovial sarcoma expressing MAGE-A4 (Hong *et al.* 2023). Furthermore, the rapid advancement of protein-based therapies has been significantly fueled by the broad repertoire of antigens targetable for TCRs, as exemplified by Kimmtrak (tebentafusp), the first bispecific TCR drug approved for metastatic or unresectable uveal melanoma (Middleton *et al.* 2020). These developments highlight the transformative potential of TCR-based strategies in oncology. Despite challenges such as tumor heterogeneity and the immunosuppressive microenvironment in the treatment of solid tumors, TCR-T therapy offers a compelling approach to overcoming these obstacles. With its ability to recognize a wide range of antigens and its potent anti-tumor effects, TCR-T therapy holds considerable potential for clinical translation (Golikova *et al.* 2024).

The identification of tumor-specific TCRs is a critical step in TCR-T-based cancer immunotherapy. Multiple strategies have been developed to isolate TCRs with high affinity and specificity, each leveraging distinct experimental approaches: (1) Co-culturing of patient-derived PBMCs or TILs with tumor antigens, followed by the application of MHC-peptide complexes (pHLA multimers) to isolate TCRs capable of recognizing target antigens with precision (Labarrière *et al.* 2008); (2) Stimulation of healthy donor PBMCs using *ex vivo* matured dendritic cells (DCs) loaded with tumor-associated antigens or peptides to activate and expand antigen-specific T cells (Carreno *et al.* 2015); (3) Isolation of antigen-specific T cells after *in vivo* vaccination or peptide immunization in patients, with subsequent screening for reactive TCRs using flow cytometry or functional assays (Chen *et al.* 2022); (4) Immunization of transgenic or humanized mice expressing human HLA molecules with human tumor antigens to generate high-affinity T cell responses, followed by TCR cloning, screening, and validation for therapeutic applications (Salguero *et al.* 2014, Zhang *et al.* 2021).

The methods mentioned above offer distinct advantages and limitations. Patient-derived PBMCs and TILs are highly clinically relevant but are constrained by limited sample availability and the functional state of the cells (Poschke *et al.* 2020). While approaches utilizing healthy donor-derived PBMCs are more readily accessible and facilitate broader antigen screening, they may result in the identification of TCRs with suboptimal affinity for tumor antigens (Tubb *et al.* 2018). Furthermore, tumor-specific T cells in healthy donors are usually rare and present at low frequencies, making the screening process less cost-effective (Ali *et al.* 2022). Animal models provide a valuable platform for generating high-affinity TCRs, particularly for rare antigens or neoantigens, but they require optimization for better alignment with human immunology (Wang *et al.* 2016). Ultimately, selecting the most appropriate strategy requires careful consideration of specific experimental objectives and the resources available within a given laboratory setting.

To enable the rapid and efficient screening of antigen-specific TCRs, we developed a protocol to isolate TCRs from immunized HLA-transgenic mice using an optimized single-cell sequencing method (Zhang *et al.* 2021). The protocol begins with the identification of epitopes from tumor cell lines through mass spectrometry. The identified peptides are then used to immunize HLA-transgenic mice, enriching for antigen-specific T cells. Antigen-specific T cells are isolated from the spleens of immunized mice via single-cell sorting, followed by reverse transcription and nested PCR to rapidly amplify the variable (V) gene sequences of the TCRs. Finally, the TCRs are validated through binding assays and functional verifications to confirm their specificity for the antigen and their cytotoxicity.

**Figure 1:**
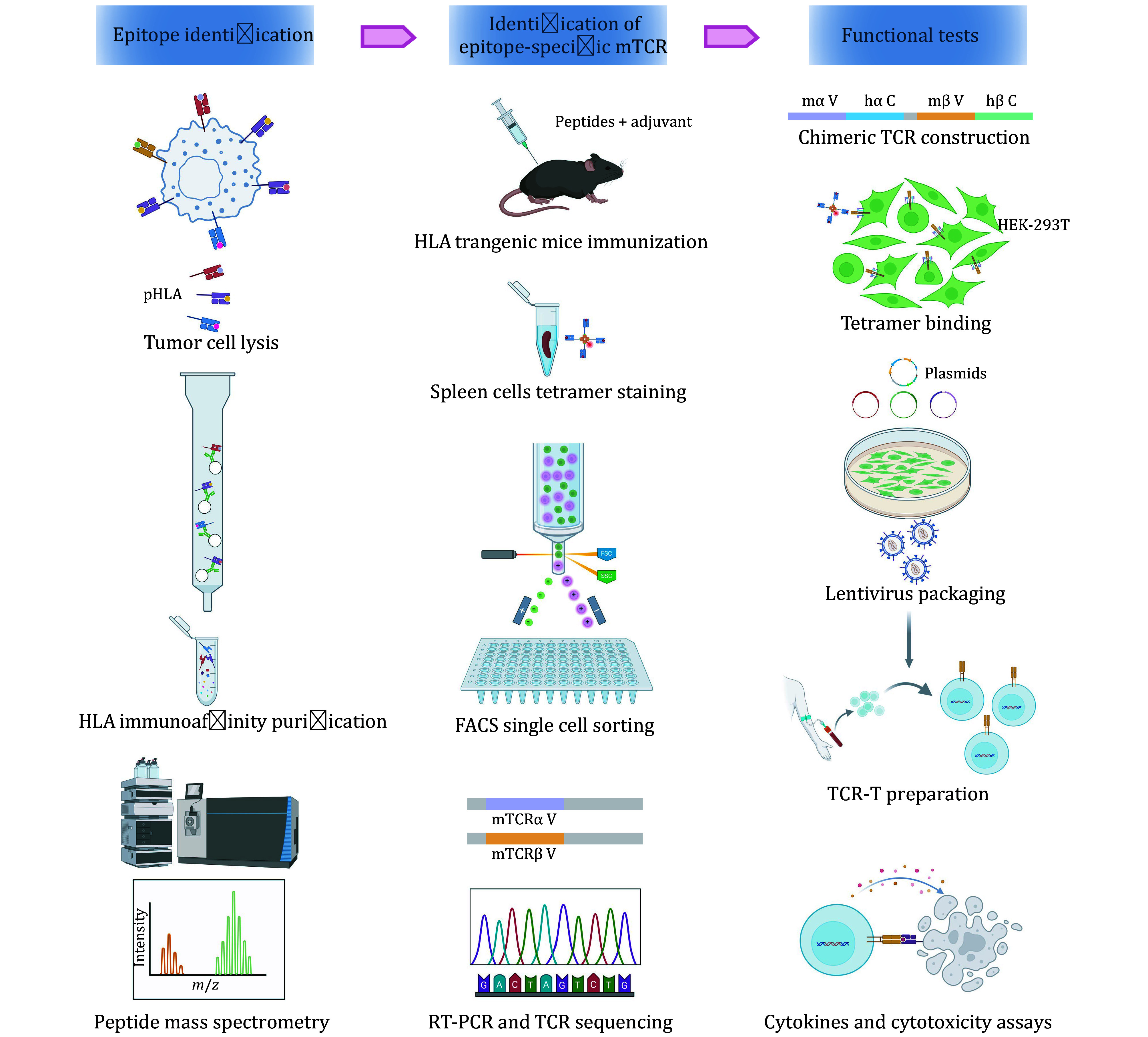
The workflow of the identification of immunopeptide and antigen-specific TCR. A schematic overview of the key steps in the protocol, comprising three main sections: (1) epitope identification (Step 1), (2) Identification of epitope-specific mTCR (Steps 2 to 3), and (3) functional tests (Step 4)

This efficient approach enables the rapid completion of the entire workflow from epitope identification to TCR screening and *in vitro* functional validation (Fig. 1). In conclusion, this protocol provides a rapid and efficient platform for preliminary TCR screening, and lays a strong foundation for advancing the clinical translation of TCR-based therapies.

## STEP-BY-STEP PROCEDURE

### Step 1: Identification tumor antigen associated epitopes by MS/MS

Tumor cells process self-antigen peptides and present them on their surface through HLA molecules, enabling subsequent immune recognition. To identify these processed epitopes, membrane pHLA proteins are isolated and enriched from tumor cells via immunoprecipitation. The selection of cell lines should ensure the expression of both the target HLA and antigen. The bound peptides are subsequently eluted from the antigen-binding grooves of the HLA molecules and subjected to mass spectrometry analysis to characterize their sequences and quantify their relative abundances. Since CD8^+^ cytotoxic T lymphocytes (CTLs) play a key role in tumor cell elimination and HLA class I molecules (HLA-ABC) specifically interact with CD8^+^ T cells, this protocol is primarily designed for the identification and characterization of peptides presented by HLA class I molecules. The tumor antigens investigated in TCR-T clinical trials are summarized in Table 1. These classical antigens can be candidates for research.

#### Step 1.1: Preparation of immunoaffinity column [TIMING 3 h]

##### Materials

• W6/32 antibody (Abcam, Cat. ab22432)

• Protein-A Sepharose (Thermo Scientific, Cat. 101041)

• Econo-Column (Bio-Rad, Cat. 7374006)

• PBS (Beyotime, Cat. C0221A)

• Borate buffer (Sigma, Cat. 89273-100ML)

• Triethanolamine (TEO; Merck, Cat. 102-71-6)

• Cross-link buffer: 40 mmol/L dimethyl pimelimidate dihydrochloride (DMP-2HCl; Sigma, Cat. D8388-250MG) in 0.2 mol/L TEO, pH 8.3

• Terminal buffer: 0.2 mol/L Tris (Sigma, Cat. 77-86-1), pH 8.0

• Stripping buffer: 0.1 mol/L citrate buffer (Thermo Scientific, Cat. 005000), pH 3.0

**Table 1:** Targeted antigens and epitopes in TCR-T clinical trials

Antigen type	Target antigen	Epitope	HLA restriction	Reference
TDAs	MART-1	AAGIGILTV	HLA-A*02:01	Morgan *et al.* 2006
	MART-1	EAAGIGILTV	HLA-A*02:01	Chodon *et al.* 2014
	gp100	KTWGQYWQV	HLA-A*02:01	Johnson *et al.* 2009
	CEA	IMIGVLVGV	HLA-A*02:01	Parkhurst *et al.* 2011
TGAs	MAGE-A3	KVAELVHFL	HLA-A*02:01	Morgan *et al.* 2013
	MAGE-A3	EVDPIGHLY	HLA-A*01	Linette *et al.* 2013
	MAGE-A3	KKLLTQHFVQENYLEY	HLA-DPB1*0401	Lu *et al.* 2015
	MAGE-A4	NYKRCFPVI	HLA-A*24:02	Kageyama *et al.* 2015
	MAGE-A4	GVYDGREHTV	HLA-A*02	Blum Murphy *et al.* 2023
	MAGE-A10	GLYDGMEHL	HLA-A*02:01 or HLA-A*02:06	Lam *et al.* 2018
	PRAME	SLLQHLIGL	HLA-A*02	Wermke *et al.* 2023
	NY-ESO-1	SLLMWITQC	HLA-A*02:01	Robbins *et al.* 2011
Viral antigens	HPV16-E6	TIHDIILECV	HLA-A*02:01	Doran *et al.* 2019
	HPV16-E7	YMLDLQPET	HLA-A*02:01	Nagarsheth *et al.* 2021
	HBV	N.A.	HLA-A*02:01 or HLA-C*08:01	Meng *et al.* 2021
	MCPyV	KLLEIAPNC	HLA-A*02:01	Veatch *et al.* 2022
Neo-antigens	TP53 R175H	HMTEVVRHC	HLA-A*02:01	Hsiue *et al.* 2021
	KRAS G12D	GADGVGKSA	HLA-C*08:02	Leidner *et al.* 2022
TDAs: Tissue differentiation antigens; TGAs: Tumor testis antigens				

##### Main steps

Step 1.1.1: Transfer 1 mL Protein-A Sepharose into a 50 mL tube. Wash the resin twice by adding 5 column volumes (CV) of PBS and centrifuging 4000*g*, 2 min.

Step 1.1.2: Dilute W6/32 antibody to 1–10 mg/mL with PBS. Add 2 mg of antibody to the resin. Rotate gently at 4°C for 40 min.

Step 1.1.3: Transfer the mixture to an Econo-Column and allow the antibody to flow through by gravity. Wash the resin with 10 CV of 0.1 mol/L borate buffer (pH 8.0), followed by 10 CV of 0.2 mol/L triethanolamine (pH 8.2).

Step 1.1.4: Pass 5 CV of cross-link buffer through the column to initiate the cross-linking reaction. Let the cross-link buffer flow until there is a small meniscus above the resin. Seal the bottom of the column, and incubate at RT for 1 h.

Step 1.1.5: Wash the resin with 10 CV of terminal buffer to terminate the reaction.

Step 1.1.6: Remove unbound antibody with 10 CV of stripping buffer.

Step 1.1.7: Pass 10 CV of PBS to equilibrate the column.

#### Step 1.2: HLA immunoaffinity purification from cell lysate [TIMING 3 h]

##### Materials

• PBS

• 1× lysis buffer: 0.5% (*v*/*v*) IGEPAL CA-630 detergent (Sigma, Cat. I8896), 1:200 of protease inhibitor cocktail (Roche, Cat. 11836145001), in PBS

• Elution buffer: 0.1 mol/L acetic acid (Sigma, Cat. 64-19-7)

• Trifluoroacetic Acid (TFA; Thermo Scientific, Cat. 85183)

• Sample: tumor cell lines

• Centrifuge concentrator (Eppendorf, Cat. 07-748-13)

##### Main steps

Step 1.2.1: Culture the cell lines of interest to 5 × 10^7^–5 × 10^8^. Wash cells twice with PBS and harvest by scraping the adherent cells after adding 2 mL of 1× lysis buffer. Incubate the lysed sample for 45 min at 4°C with gentle rotation. Centrifuge the sample for 1 h at 12,000*g*, 4°C.

Step 1.2.2: Flow the supernatant through an equilibrate protein A column to remove endogenous antibodies. Load the cleared lysate directly onto the immunoaffinity column (Step 1.1.7) and incubate for 1 h at 4°C.

Step 1.2.3: Pass the lysate and wash the immunoaffinity column with 10 CV of PBS to remove detergent thoroughly.

Step 1.2.4: Elute the pHLA by adding 5 CV of elution buffer then vacuum concentrate the sample and reconstitute the sample into 0.1% (*v*/*v*) TFA.

#### Step 1.3: Separation of HLA peptides by RP-HPLC [TIMING 1.5 h]

##### Materials

• Formic acid (FA; Sigma, Cat. 64-18-6)

• Buffer A: 0.1% (*v*/*v*) TFA in MS-grade water

• Buffer B: 0.1% (*v*/*v*) TFA in MS-grade 80% (*v*/*v*) acetonitrile (Sigma, Cat. 75-05-8)

• C18 column (Merck, Cat. 102129000)

• LoBind microcentrifuge tubes (Eppendorf, Cat. EPPE0030108.132)

• AKTA micro flow HPLC (Cytiva)

• Centrifuge concentrator

##### Main steps

Step 1.3.1: Equilibrate C18 column in buffer A.

Step 1.3.2: Load the sample onto the C18 column with a gradient of 2% buffer B. Run the following gradient: 0%–15% buffer B over 0.25 min, 15%–30% buffer B over 4 min, 30%–40% buffer B over 8 min, 40%–45% buffer B over 10 min, and 45%–99% buffer B over 2 min. Fractionate the eluate into LoBind tubes.

Step 1.3.3: Collect and concatenate the peptide-containing fractions into a total of ten fractions. Vacuum concentrate and reconstitute each in 12–15 μL of 0.1% FA.

#### Step 1.4: Mass spectrometry and data analysis [TIMING 16+ h]

##### Materials

• Buffer A: 0.1% (*v*/*v*) formic acid (FA) in MS-grade water

• Buffer B: 0.1% (*v*/*v*) FA in MS-grade 80% (*v*/*v*) acetonitrile

• C18 trap column (Thermo Scientific, Cat. 164199)

• Analysis software: ProteinPilot or PEAKS

##### Main steps

Step 1.4.1: Load the samples onto the C18 trap column at 15 μL/min.

Step 1.4.2: Elute and separate peptides on the in-line analytical column at a flow rate of 250 μL/min using a 101-min linear gradient of 2.5%–99% of buffer B in buffer A. The gradient starts from 2.5% buffer B and increases to 7.5% buffer B in 1 min; this is followed by a linear gradient to 37.5% buffer B for 90 min and then an increase to 99% buffer B in 10 min.

Step 1.4.3: Analyze MS and MS/MS data using specialized software. For data comparison, perform searches using a non-enzymatic or non-specific enzymatic digestion approach against a comprehensive human protein database, including the target antigen (*e*.*g*., mutant protein), to enhance identification accuracy and specificity. Synthesize peptides of interest.

### Step 2: Identification of immunogenic peptides through immunization of HLA transgenic mice

Naturally processed epitopes identified by mass spectrometry may not exhibit immunogenicity *in vivo*. Here, we outline a protocol involving three rounds of peptide immunization in HLA-transgenic mice, followed by ELISPOT assays to evaluate peptide immunogenicity. Refolded pHLA proteins are subsequently generated from inclusion bodies, facilitating tetramer preparation (Jiang *et al.* 2022) for downstream flow cytometric screening of antigen-specific TCRs.

#### Step 2.1: Immunization of HLA transgenic mice [TIMING 3 w]

##### Materials

• Peptides (Synthesized at GenScript, purity > 95%)

• Dimethyl sulfoxide (DMSO; Sigma, Cat. 67-68-5)

• PBS

• T cell adjuvant Quick CTL (Biodragon, Cat. KX0210044)

• Insulin syringes (BD biosciences, Cat. 328421)

• Animal: 8–12 week HLA transgenic mice (Purchase from Biocytogen or Jackson lab)

##### Main steps

Step 2.1.1: Dissolve the peptides in DMSO to 50 mg/mL. Add 2 μL of each peptide to 50 μL of T cell adjuvant and supplement the volume with PBS to 100 μL.

Step 2.1.2: Immunize HLA transgenic mice subcutaneously at the inguinal region with peptide reagent (Step 2.1.1) or 100 μL of PBS as negative control.

Step 2.1.3: Immunize once a week for a total of three times. One week after the last immunization, euthanize the mice and obtain their spleens in a sterile biosafety cabinet.

#### Step 2.2: Identification of immunogenic peptides by ELISPOT assay [TIMING 2 d]

##### Materials

• 70 µm cell strainer (Falcon, Cat. 352350)

• Peptides

• Mouse IFN-γ ELISPOT kit (Mabtech, Cat. 3321-4HPW-10)

• PMA + Ionomycin (Dakewe Bioengineering, Cat. 2030421)

• Mouse spleen cells

• ACK lysis buffer (Thermo Scientific, Cat. A1049201)

• RPMI 1640 (Gibco, Cat. 11875093)

• Fetal bovine serum (FBS; Gibco, Cat. 10270-106)

• Culture medium: RPMI 1640 + 10% FBS

• Freezing medium: 90% FBS + 10% DMSO

##### Main steps

Step 2.2.1: Prepare mice single-cell suspensions by grinding spleens on a 70-µm cell strainer in a biosafety cabinet. Centrifuge 400*g*, 5 min, and discard the supernatant.

Step 2.2.2: Add 3 mL of ACK lysis buffer and incubate the cells for 5 min at 4°C. Wash the cells twice by adding 5 mL of RPMI 1640 and centrifuging 400*g*, 5 min. Resuspend the cells in a culture medium at a density of 2 × 10^6^ viable cells/mL.

Step 2.2.3: Perform ELISPOT assay by adding 100 μL of spleen cells and 100 μL of peptide (20 μg/mL) to a pre-coated ELISPOT 96-well plate. In the negative control well, replace the peptide with the same volume of culture medium. In the positive control well, replace the peptide with diluted PMA (500 ng/mL) + Ionomycin (10 µg/mL).

Step 2.2.4: Wash the remaining cells twice with PBS and resuspend them in a freezing medium at a final concentration of 2 × 10^7^ cells/mL. Aliquot 1 mL of the cell suspension into cryovials and place them in a controlled-rate freezing container to ensure gradual cooling. Transfer the vials to a −80°C freezer for short-term storage, not exceeding two weeks.

Step 2.2.5: Incubate the plate for 18 h in a 5% CO₂ cell incubator, 37°C. Process the remaining steps according to the instructions of the manufacturer.

Step 2.2.6: Analyze the results and determine the spot-forming cells (SFCs) using an automatic ELISPOT reader and image analysis software (Cellular Technology Ltd., Ohio). Set the threshold for a positive response at two times the SFC count in the negative control. Peptides generating higher SFCs are considered capable of inducing T-cell immune responses in mice.

#### Step 2.3: Preparation of pHLA tetramer for FACS [TIMING 3 d]

##### Materials

• Inclusion bodies: human β_2_m and HLA-avi

• Biotin protein ligase (GeneCopoeia, Cat. BI001)

• 10 kDa ultrafiltration concentrator (Merck, Cat. UFC9010)

• Streptavidin from Streptomyces avidinii (Sigma, Cat. 9013-20-1), 20 mg/mL in PBS

• PE Streptavidin (BD biosciences, Cat. 554061)

• Phenylmethanesulfonyl fluoride (PMSF; Sigma, Cat. 329-98-6)

• Equilibration buffer: 20 mmol/L Tris, 50 mmol/L NaCl, 0.5 mmol/L PMSF, pH 8.0

• Superdex 200 increase 10/300 GL (Cytiva, Cat. 28-9909-44)

• AKTA-pure system (Cytiva)

##### Main steps

Step 2.3.1: Construct HLA-avi gene by fusing the extracellular domain of the HLA heavy chain with an Avi-tag (Beckett *et al.* 1999) on C terminal. The amino acid sequences of HLA molecules can be retrieved from the IPD-IMGT/HLA Database (https://www.ebi.ac.uk/ipd/imgt/hla/). Optimize the human β_2_-microglobulin (hβ_2_m) gene and HLA-avi gene for codon usage in *E. coli* and synthesize the genes.

Step 2.3.2: Isolate the hβ_2_m inclusion bodies and HLA-avi inclusion bodies. Refold HLA-avi and hβ_2_m with the synthesized peptide, then purify the soluble pHLA-avi complex using a Superdex 200 Increase 10/300 GL column equilibrated with the equilibration buffer. The pHLA-avi complex elutes at 15.5 mL. SDS-PAGE analysis should confirm the presence of a ~35 kDa heavy chain band and an ~11 kDa light chain band, indicating successful refolding and complex formation.

Step 2.3.3: Biotinylate the pHLA-avi protein overnight at 4°C using biotin protein ligase, according to the manufacturer’s protocol, followed by purification via gel filtration.

Step 2.3.4: Concentrate the pHLA-avi protein to approximately 2 mg/mL using a 10 kDa ultrafiltration concentrator. Prepare the following three samples: (1) Sample A: 8 μL pHLA-avi protein + 2 μL equilibration buffer; (2) Sample B: 8 μL pHLA-avi protein + 2 μL streptavidin; (3) Sample C: 8 μL equilibration buffer + 2 μL streptavidin.

Step 2.3.5: Perform a streptavidin-shift assay using SDS-PAGE to assess the efficiency of *in vitro* biotinylation of the pHLA-avi protein. Calculate the biotinylation efficiency using the following formula:

Efficiency = (Band intensity at 35 kDa in Sample A − Band intensity at 35 kDa in Sample B)/ Band intensity at 35 kDa in Sample A.

Step 2.3.6: Mix the biotinylated pHLA-avi protein with PE streptavidin to form pHLA tetramer.

### Step 3: Single cell isolation and TCR sequencing by RACE

**Figure 2:**
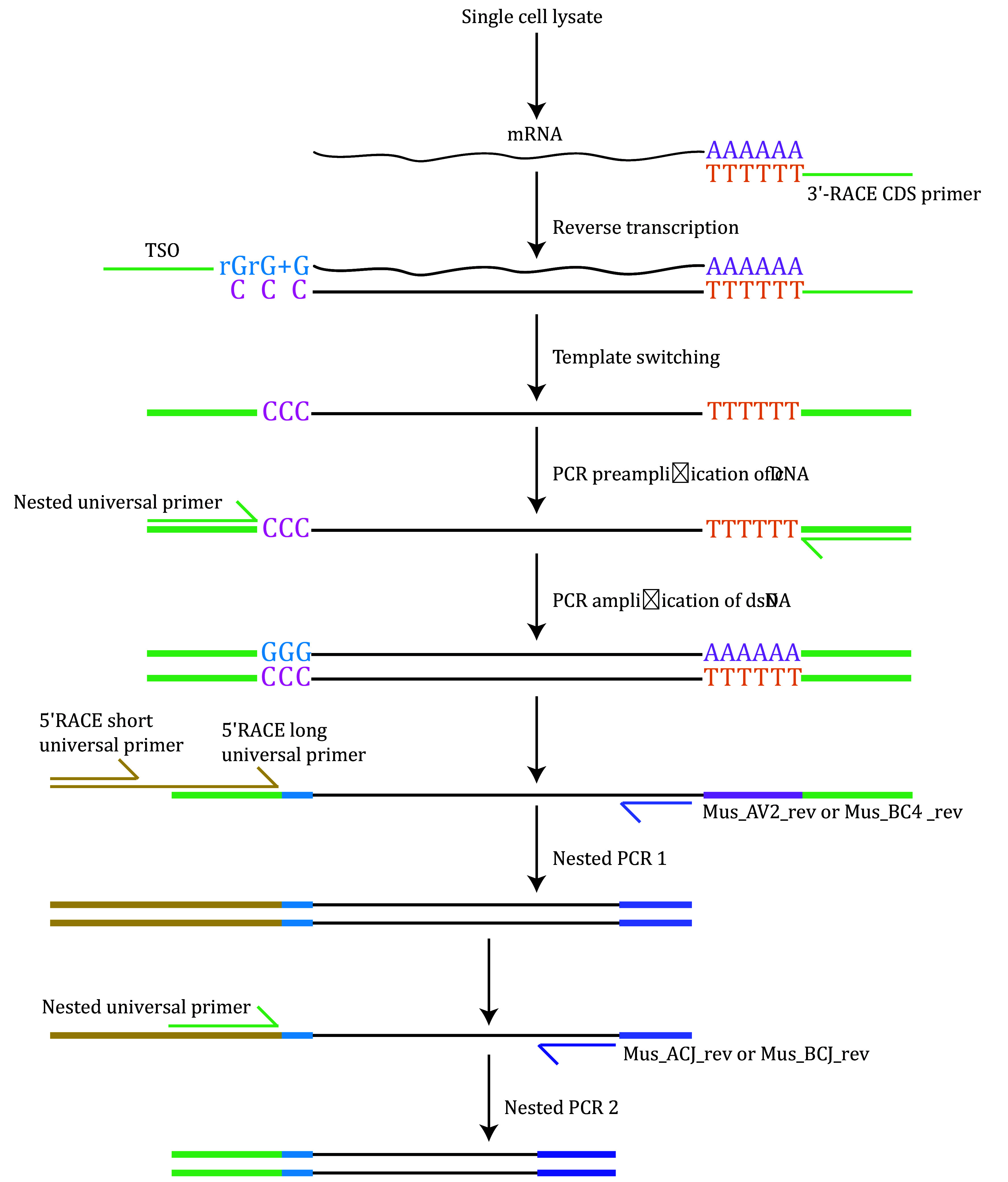
Flowchart for single-cell TCR sequencing. The outline of the TCR sequencing protocol is detailed from Step 3.1 to Step 3.4, encompassing the key procedures for reverse transcription and nested PCR. The primers used for each step are listed in Table 2

To isolate antigen-specific T cells and identify paired TCR sequences, we employ flow cytometry (FACS)-based single-cell sorting, followed by reverse transcription and nested PCR. This approach enables the efficient amplification of TCR V-region DNA fragments from mouse T cells, which are seamlessly cloned into linear vectors for sequencing. V(D)J gene rearrangement is subsequently analyzed using IMGT. Given that antigen recognition induces clonal expansion, TCR sequences with higher frequencies in the sequencing data are prioritized as likely antigen-specific candidates. These high-frequency TCRs are selected for downstream binding assays and functional validation (Fig.2).

#### Step 3.1 Detection and single cell isolation tetramer-positive splenocytes by FACS [TIMING 3 h]

##### Materials

• Culture medium: RPMI 1640 + 10%FBS

• Fixable viability stain 780 (BD biosciences, Cat. 565388)

• pHLA-tetramer-PE (Step 2.3)

• FITC anti-mouse CD3 Antibody (Biolegend, Cat. 100204)

• PerCP/Cyanine5.5 anti-mouse CD8a Antibody (Biolegend, Cat. 100734)

• Recombinant RNase inhibitor (Takara, Cat. 2313B)

• DEPC-treated water (Thermo scientific, Cat. R0601)

• Triton X-100 (Sigma, Cat. X100PC)

• dNTP mix (Takara, Cat. 639132)

• 96-well PCR plate (Bio-rad, Cat. MLP9601)

• Primers: listed in Table 2, synthesized in Generay

**Table 2:** Primers used in this protocol

Primer	Application	Sequence
3'-RACE CDS primer	Reverse transcription	AAGCAGTGGTATCAACGCAGAGTAC(T)30 V N
TSO	Template switching	AAGCAGTGGTATCAACGCAGAGTACATrGrG+G
Nested universal primer	Double strand DNA amplification, nested Primer	AAGCAGTGGTATCAACGCAGAGT
5'RACE long universal primer	5'RACE Primer 1, incorporation of suppression PCR inverted repeat elements	CTAATACGACTCACTATAGGGCAAGCAGTGGTATCAACGCAGAGT
5'RACE short universal primer	5'RACE Primer 2	CTAATACGACTCACTATAGGGC
Mus_AV2_rev	Nested primer 1, mouse TCR alpha library	GGTGCTGTCCTGAGACCGAG
Mus_BC4_rev	Nested primer 1, mouse TCR beta library	GATGGCTCAAACAAGGAGACC
Mus_ACJ_rev	Nested primer 2, mouse TCR alpha library	CAGGTTCTGGGTTCTGGATGT
Mus_BCJ_rev	Nested primer 2, mouse TCR beta library	GGAGTCACATTTCTCAGATCCT

• FACS Aria III (BD Biosciences)

##### Main steps

Step 3.1.1: Refresh mouse splenocytes and culture them overnight at 37°C in a culture medium.

Step 3.1.2: On the next day, prepare cell lysis buffer in a biosafety cabinet that contains 2.5% of Recombinant RNase inhibitor, 0.1% of Triton X-100, 2.5 μmol/L of 3'-RACE CDS Primer A, 2.5 mmol/L of dNTP mix in 96-well PCR plate (4 μL/well).

Step 3.1.3: Stain splenocytes with fixable viability stain 780 at RT for 20 min.

Step 3.1.4: Mix FITC anti-mouse CD3 antibody, PerCP/Cyanine5.5 anti-mouse CD8a antibody, and pHLA-tetramer-PE with splenocytes and incubate at RT for 30 min.

Step 3.1.5: Wash the splenocytes twice with PBS to remove unbound antibodies.

Step 3.1.6: Single-cell sort CD3^+^/CD8^+^/pHLA-tetramer^+^ live T cells into 96-well PCR plate (Step 3.1.2) on a FACS Aria sorter.

#### Step 3.2: Reverse transcriptase-PCR [TIMING 3 h]

##### Materials

• DEPC-treated water

• 5× M-MLV Reverse Transcriptase Buffer (Thermo scientific, Cat. 18057018)

• M-MLV reverse transcriptase (Thermo scientific, Cat. 28025013)

• Recombinant RNase inhibitor

• Betaine (Sigma, Cat. 61962)

• MgCl_2_ (Thermo scientific, Cat. F-5101)

• Primers: listed in Table 2

• Thermal cycler

##### Main steps

Step 3.2.1: Spin down the samples (700*g* for 10 s at RT). Incubate the samples at 72°C for 3 min and 42°C for 2 min in thermal cycler.

Step 3.2.2: Spin down the samples, and then put them immediately back on ice. Prepare the reverse transcriptase mix by mixing the reagents listed in Table 3.

**Table 3:** Components for preparing the reverse transcriptase mix

Component	Volume (μL)
5× M-MLV Reverse Transcriptase Buffer	2
RNAse inhibitor (40 U/µL)	0.25
Betaine (5 mol/L)	2
MgCl_2_ (50 mmol/L)	0.6
TSO (20 µmol/L)	0.5
M-MLV reverse transcriptase	0.5
H_2_O	0.2
Total	6.05

Step 3.2.3: Add 6.05 µL of the reverse transcriptase mix to the samples from Step 3.2.1 to obtain a final reaction volume of 10 µL.

Step 3.2.4: Spin down the samples, and incubate the reaction in a thermal cycler with a heated lid, as detailed in Table 4.

**Table 4:** Details for the thermal cycler of Step 3.2.4

Cycle	Temperature (°C)	Time (min)
1	42	90
2-11	50	2
	42	2
12	70	15
13	4	Hold

#### Step 3.3: Double strand cDNA amplification [TIMING 2 h]

##### Materials

• DEPC-treated water

• Phusion PCR Mix (Thermo scientific, Cat. F531L)

• Primers: listed in Table 2

• Thermal cycler

##### Main steps

Step 3.3.1: Spin down the samples, and then put them immediately back on ice. Prepare the PCR mix by mixing the reagents listed in Table 5.

**Table 5:** Components for preparing the PCR mix

Component	Volume (μL)
H_2_O	14
Phusion PCR Mix	25
Nested universal primer	1
Total	40

Step 3.3.2: Add 40 µL of the PCR mix to the samples from Step 3.2.4 to obtain a final reaction volume of 50 µL.

Step 3.3.3: Spin down the samples, and incubate the reaction in a thermal cycler with a heated lid, as detailed in Table 6.

**Table 6:** Details for the thermal cycler of Step 3.3.3

Cycle	Temperature (°C)	Time
1	98	30 s
2-31	98	10 s
	65	30 s
	72	90 s
32	72	10 min
33	4	Hold

#### Step 3.4: Nested PCR [TIMING 5 h]

##### Materials

• DEPC-treated water

• Phusion PCR Mix

• Primers: listed in Table 2

• Thermal cycler

• Quick Gel Extraction Kit (Qiagen, Cat. 28704)

Main steps

Step 3.4.1: Prepare 10× UPM by mixing the reagents listed in Table 7.

**Table 7:** Components for preparing the 10× UPM

Component	Volume (μL)
H_2_O	19
5'RACE long universal primer (10 µmol/L)	1
5'RACE short universal primer (10 µmol/L)	5
Total	25

Step 3.4.2: Spin down the samples, and then put them immediately back on ice. Prepare the nested PCR template 1 by adding 5 µL of the samples to 200 µL of DEPC-treated water.

Step 3.4.3: Prepare the nested-PCR mix-1 by mixing the reagents listed in Table 8.

**Table 8:** Components for preparing the nested-PCR mix-1

Component	Volume (μL)
H_2_O	5.6
Phusion PCR Mix	10
10× UPM	2
Mus_AV2_rev (10 µmol/L) for α chain Or Mus_BC4_rev (10 µmol/L) for β chain	0.4
Total	18

Step 3.4.4: Mix 18 µL of the nested-PCR mix-1 and 2 µL of nested PCR template 1 from Step 3.4.2 to obtain a final reaction volume of 20 µL.

Step 3.4.5: Spin down the samples, and incubate the reaction in a thermal cycler with a heated lid, as detailed in Table 9.

**Table 9:** Details for the thermal cycler of Step 3.4.5

Cycle	Temperature (°C)	Time
1	98	30 s
2-31	98	10 s
	65	30 s
	72	30 s
32	72	10 min
33	4	Hold

Step 3.4.6: Spin down the samples, and then put them immediately back on ice. Prepare the nested PCR template 2 by adding 5 µL of the samples to 125 µL of DEPC-treated water.

Step 3.4.7: Prepare the nested-PCR mix-2 by mixing the reagents listed in Table 10.

**Table 10:** Components for preparing the nested-PCR mix-2

Component	Volume (μL)
H_2_O	7.2
Phusion PCR Mix	10
Nested universal primer (10 µmol/L)	0.4
Mus_ACJ_rev (10 µmol/L) for α chain Or Mus_BCJ_rev (10 µmol/L) for β chain	0.4
Total	18

Step 3.4.8: Mix 18 µL of the nested-PCR mix-2 and 2 µL of nested PCR template 2 from Step 3.4.6 to obtain a final reaction volume of 20 µL.

Step 3.4.9: Spin down the samples, and incubate the reaction in a thermal cycler with a heated lid, as detailed in Table 11.

**Table 11:** Details for the thermal cycler of Step 3.4.9

Cycle	Temperature (°C)	Time
1	98	30 s
2-26	98	10 s
	65	30 s
	72	30 s
27	72	10 min
28	4	Hold

Step 3.4.10: Purify PCR products using 1% agarose gel electrophoresis and following the manufacturer of Qiagen quick Gel Extraction Kit.

#### Step 3.5: Transformation and TCR sequencing [TIMING 3+ d]

##### Materials

• pEASY-Blunt Zero Cloning Kit (TransGen, Cat. CB501-01)

• Trans5α Chemically Competent Cell (TransGen, Cat. CD201-01)

• LB medium (Thermo Scientific, Cat. 10855001)

• pCDH-EF1α-MCS-T2A-Puro (System Biosciences, Cat. CD520A-1)

• Water bath

##### Main steps

Step 3.5.1: Prepare in-fusion cloning reagents listed in Table 12.

**Table 12:** Components for preparing the in-fusion cloning reagents

Component	Volume (μL)
H_2_O	1
PCR product	3
Cloning vector	1
Total	5

Step 3.5.2: Spin down the samples, and incubate the reaction at 25°C for 15 min.

Step 3.5.3: Add the ligated products to 50 μL of Trans5α Chemically Competent Cell, and incubate on ice for 30 min.

Step 3.5.4: Heat-shock the cells at 42°C for 30 s, then immediately place the tube on ice for 2 min.

Step 3.5.5: Add 300 μL of LB medium to the tube and shake the tube at 37°C (200 r/min) for 1 h.

Step 3.5.6: Centrifuge the cells for 1 min at 1000*g*, 25°C. Resuspend the cells in 100 μL LB medium and spread them on ampicillin-resistant s plates. Incubate at 37°C overnight.

Step 3.5.7: Analyze the sequences by sequencing with M13F and M13R primers. Obtain TCR VDJ gene rearrangement information on IMGT website.

Step 3.5.8: Design chimeric TCR by combining the mouse TCR V region with the human TCR C region, and clone it into the pCDH lentiviral vector.

### Step 4: Functional assays in primary T cells

To minimize potential false positives arising from flow cytometric sorting, cloned TCRs are first validated for specific binding to pHLA tetramers. TCR plasmids are transfected into 293T cells, and TCR-pHLA binding is assessed using flow cytometry. Lentiviral vectors encoding the TCRs are then produced and used to transduce primary T cells, generating TCR-T cells. The functionality of the TCRs is evaluated through *in vitro* cytokine secretion assays and cytotoxicity assays.

#### Step 4.1: Binding assay of TCR with pHLA-tetramer in HEK-293T cells system [TIMING 2 d]

##### Materials

• HEK-293T cells (ATCC, Cat. CRL-3216)

• pHLA-tetramer-PE (Step 2.3)

• APC anti-human CD3 Antibody (Biolegend, Cat. 300412)

• PerCP/Cyanine5.5 anti-human TCR α/β Antibody ((Biolegend, Cat. 306724)

• Chimeric TCR plasmid (Step 3.5.8)

• Human CD3 plasmid (Synthesize in Genscript)

• Polyethylenimin (PEI; Polysciences, Cat. 23966-100)

• Opti-MEM (Gibco, Cat. 31985070)

• DMEM (Gibco, Cat. C11965500BT)

• FBS

##### Main steps

Step 4.1.1: Seed HEK-293T cells in a 6-well plate and culture them overnight in DMEM containing 10% FBS. On the following day, perform plasmid transfection when cells are about 70% confluent.

Step 4.1.2: Add 2 μg of human CD3 plasmid and 2 μg of chimeric TCR plasmid to 100 μL of Opti-MEM, add 12 μg of PEI to 100 μL of opti-MEM in another tube. Incubate 5 min at RT.

Step 4.1.3: Mix the plasmids and PEI contained in Step 4.1.2 together. After a 20-minute incubation, add 200 μL of the plasmid-PEI directly to each well containing HEK-293T cells.

Step 4.1.4: Remove the cell culture supernatant 4 h after transfection and replace it with DMEM containing 2% FBS. After 24 h of culture, detach the cells for flow cytometry analysis.

Step 4.1.5: Wash the cells once with PBS by centrifuging for 5 min at 400*g*, 25°C. Then stain the cells with pHLA-tetramer, APC anti-human CD3 antibody and PerCP/Cyanine5.5 anti-human TCR α/β antibody at RT for 30 min.

Step 4.1.6: Wash the cells twice with PBS by centrifuging for 5 min at 400*g*, 25°C. Resuspend the cells with 200 μL of PBS, then analyze on flow cytometry.

#### Step 4.2 Packaging TCR lentivirus [TIMING 3 d]

##### Materials

• HEK-293T cells

• Chimeric TCR plasmid (Step 3.5.8)

• pLP1 plasmid (Addgene, Cat. 209988)

• pLP2 plasmid (Addgene, Cat. 209989)

• pCMV-VSV-G plasmid (Addgene, Cat. 8454)

• PEI

• Opti-MEM

• Sodium butyrate (MyBioSouce, Cat. MBS6101864)

• 45 μm PES filter (Merck, Cat. SLHPM33RS)

• 100 kDa ultrafiltration concentrator (Merck, Cat. UFC910008)

##### Main steps

Step 4.2.1: Seed HEK-293T cells in 15-cm dishes and culture them overnight. On the following day, perform plasmid transfection when cells are about 90% confluent.

Step 4.2.2: Add 20 μg of chimeric TCR plasmid, 20 μg of pLP1 plasmid, 13 μg of pLP2 plasmid and 5 μg of VSVG plasmid to 1 mL of Opti-MEM, add 174 μg of PEI to 1 mL of opti-MEM in another tube. Incubate for 5 min at RT.

Step 4.2.3: Mix the plasmids containing Opti-MEM with the PEI containing opti-MEM together. After a 20-min incubation, add 2 mL of the plasmid-PEI directly to each 15-cm dish containing HEK-293T cells.

Step 4.2.4: Remove the cell culture supernatant 6 h after transfection and replace it with DMEM containing 2% serum and 1.2 mmol/L sodium butyrate. After 48 h of culture, collect the cell supernatant and filter it using a 45 μm PES filter.

Step 4.2.5: Concentrate the TCR lentivirus supernatant 20-fold using a sterile 100 kDa ultrafiltration concentrator.

#### Step 4.3 Preparing TCR-T cells [TIMING 6+ d]

##### Materials

• Primary T cells (Isolate from the peripheral blood of healthy donors)

• TCR lentivirus (Step 4.2)

• T Cell TransAct (Miltenyi, Cat. 130-111-160)

• TexMACS™ Medium (Miltenyi, Cat. 130-097-196)

• Recombinant Human IL-2 (Novoprotein, Cat. C013)

• Recombinant Human IL-7 (Novoprotein, Cat. C086)

• T cell culture medium: TexMACS™ Medium + 300 IU/mL Recombinant Human IL-2 + 10 ng/mL Recombinant Human IL-7

• Protamine sulfate (Sigma, Cat. P3369-10G)

##### Main steps

Step 4.3.1: Seed primary T cells in a 12-well plate at a density of 1 × 10^6^ viable cells/mL in T cell culture medium, add 10 μL of T Cell TransAct to each well.

Step 4.3.2: After 24 h of activation, centrifuge the T cells for 5 min at 400*g*, and resuspend the T cells in 500 μL of T cell culture medium for each well. Add 500 μL concentrated TCR lentivirus (Step 4.2.5) and 1.2 μg of protamine sulfate to T cells.

Step 4.3.3: Centrifuge the T cell containing 12-well plate 1 h at 1000*g*, 35°C. After centrifugation, culture the T cells in a 5% CO₂ incubator.

Step 4.3.4: Add fresh T cell culture medium to the T cells every two days, maintaining a cell density of 5 × 10^5^ to 1 × 10^6^ cells/mL.

#### Step 4.4: ELISPOT cytokine secretion assay [TIMING 3 d]

##### Materials

• TCR-T cells (Step 4.3)

• Target cells: expressing the targeted antigen and corresponding HLA

• Human IFN-γ ELISPOT kit (Mabtech, Cat. 3420-2HPW-10)

• T cell rest medium: PRMI 1640 + 10% FBS

• Peptides (Synthesized at GenScript)

##### Main steps

Step 4.4.1: One day before ELISPOT assay, culture T cells in a T cell rest medium for 24 h.

Step 4.4.2: On the second day, resuspend the T cells at a density of 1 × 10^6^ viable cells/mL in the T cell rest medium. Resuspend the target cells at a density of 5 × 10^5^ viable cells/mL in the T cell rest medium.

Step 4.4.3: For ELISPOT assay with peptides, add 100 μL of T cells, 100 μL of target cells (HLA^pos^ tumor cell lines) and 2 μg of peptide (40 mg/mL) to pre-coated ELISPOT 96-well plate. In the negative control well, replace the positive peptide with negative control peptide. In the positive control well, replace the the positive peptide with diluted PMA (500 ng/mL) + Ionomycin (10 µg/mL).

Step 4.4.4: For ELISPOT assay without peptides, add 100 μL of T cells and 100 μL of target cells (HLA^pos^ and antigen^pos^ tumor cell lines) to a pre-coated ELISPOT 96-well plate. In the negative control well, replace the target cells with the same volume of culture medium. In the positive control well, replace the target cells with diluted PMA (500 ng/mL) + Ionomycin (10 µg/mL).

Step 4.4.5: Incubate for 18 h at 37°C and process the remaining steps according to the instructions of the manufacturer.

Step 4.4.6: Analyze the results and determine the SFCs using an automatic ELISPOT reader and image analysis software (Cellular Technology Ltd., Ohio). Typically, the number of SFCs in the experimental group should be at least three times higher than that in the negative control group, indicating normal cytokine secretion capability of TCR-T cells.

#### Step 4.5 Cytotoxicity assay [TIMING 2 d]

##### Materials

• TCR-T cells (Step 4.3)

• Target cells: expressing the targeted antigen and corresponding HLA

• FACS Fortessa (BD biosciences)

• T cell rest medium: PRMI 1640 + 10% FBS

• Fixable viability stain 780

• APC anti-human CD3 Antibody

• FITC anti-human CD8 Antibody (Biolegend, Cat. 344704)

• CountBright Absolute Counting Beads (Invitrogen, Cat. C36950)

##### Main steps

Step 4.5.1: One day before cytotoxicity assay, culture TCR-T cells in T cell rest medium for 24 h.

Step 4.5.2: On the second day, resuspend the TCR-T cells at a density of 1 × 10^6^ viable cells/mL in a T cell rest medium. Resuspend the target cells (HLA^pos^ and antigen^pos^ tumor cell lines) at a density of 2 × 10^5^ viable cells/mL in a T cell rest medium.

Step 4.5.3: Add 200 μL of TCR-T cells and 200 μL of target cells to each well of a 24-well plate as the experimental group. In negative control well, replace the TCR-T with Mock-T cells (T cells untransduced with TCR lentivirus). For the blank control, replace T cells with an equivalent volume of T cell rest medium.

Step 4.5.4: After 48 h of co-culture, collect all the T cells and target cells for flow cytometry analysis.

Step 4.5.5: Stain samples with fixable viability stain 780 for 20 min at RT.

Step 4.5.6: After washing the samples with PBS twice, stain the samples with a mixture of APC anti-human CD3 antibody and FITC anti-human CD8 antibody for 30 min.

Step 4.5.7: Wash the cells twice with PBS by centrifuging 5 min at 400*g*, 25°C. Resuspend the cells with 200 μL of PBS, add 10 μL of Absolute Counting Beads to each sample for standardization, and proceed with flow cytometry analysis.

Step 4.5.8: Count the tumor cells in each sample, defined as the population that does not stain positive for CD3 and CD8 fluorescent antibodies. Calculate the cytotoxicity efficiency using the following formula:

Cytotoxicity efficiency (%) = [(Tumor cell count in blank group − Tumor cell count in experimental group)/Tumor cell count in blank group] × 100.

Based on the growth rate and condition of the tumor cell lines, the expected cytotoxicity efficiency of the experimental group should range between 50%–90%, while the negative control group should not exceed 20%. If these criteria are met, the TCR can be considered to exhibit robust cytotoxic activity.

## ADVANTAGES AND LIMITATIONS OF THIS PROTOCOL

Our protocol provides a comprehensive workflow for antigen-specific TCR identification, spanning from epitope validation to functional assessment. For mass spectrometry-based peptide identification, we selected tumor cell lines due to their accessibility, genetic stability, ease of cultivation, and cost-effectiveness. Their adaptability to genetic manipulation further enhances their utility as a research model. However, these cell lines do not fully capture the biological complexity and heterogeneity of patient-derived tumors. In contrast, tumor samples from patients provide a more accurate representation of tumor heterogeneity and retain essential components of the tumor microenvironment. Despite these advantages, patient-derived samples are limited by ethical and surgical constraints, resulting in restricted availability and considerable inter-individual variability, which complicates downstream analyses. Given these factors, tumor cell lines are ideal for early-stage investigations, reproducibility studies, and the identification of immunopeptides associated with specific target antigens, whereas patient-derived tumor tissues are more suitable for studying tumor-type-specific immunopeptidomes or peptides originating from tumor-associated microbiota. Unlike other protocols that rely solely on computational predictions or basic peptide identification, we integrate multi-round peptide immunization in HLA-transgenic mice with ELISPOT assays to validate the immunogenicity of candidate epitopes. For TCR screening, we combine flow cytometry-based single-cell sorting with reverse transcription and nested PCR to isolate and amplify low-frequency antigen-specific TCR V-region sequences efficiently. Subsequently, we validate TCR specificity via pHLA tetramer binding assays in HEK-293T cells and confirm functionality through cytokine secretion and cytotoxicity assays in primary T cells. This protocol focuses on epitope identification and the rapid screening of functional TCRs, serving as an initial *in vitro* validation of TCR activity. However, since the identified TCRs are derived from mice, additional studies are required to ensure their safety before clinical application. To evaluate potential cross-reactivity, peptide library scanning can be performed to assess whether the TCR recognizes human self-proteins. Co-culture assays with normal human tissues provide further validation of off-target effects. Additionally, bioinformatics tools can be employed to predict B-cell epitopes and assess the immunogenicity of the TCR. To minimize immune responses against murine-derived TCRs, humanization of the framework regions (FRs) should be considered. These additional steps are essential for advancing candidate TCRs toward clinical translation. Overall, the advantages of our approach include high sensitivity for detecting rare T cells, robust validation of immunogenic epitopes, and reliable functional verification of TCR candidates. However, the protocol is technically demanding, and requires significant resources due to the use of transgenic mice, flow cytometry, and lentiviral transduction systems. Despite these challenges, our protocol offers a versatile and reliable strategy for identifying functionally active, antigen-specific TCRs, making it well-suited for immunological research and therapeutic applications.
